# Current Management of Traumatic Rupture of the Descending Thoracic Aorta

**DOI:** 10.2174/157340309788970324

**Published:** 2009-08

**Authors:** Riyad Karmy-Jones, Nichole Jackson, William Long, Alan Simeone

**Affiliations:** Divisions of Thoracic-Vascular and Trauma Surgery, Southwest Washington Medical Center, Vancouver WA, USA

**Keywords:** Traumatic rupture, thoracic aorta, endovascular, stent-graft.

## Abstract

Traumatic rupture of the descending thoracic aorta remains a leading cause of death following major blunt trauma. Management has evolved from uniformly performing emergent open repair with clamp and sew technique to include open repair with mechanical circulatory support, medical management and most recently, endovascular repair. This latter approach appears, in the short term, to be associated with perhaps better outcome, but long term data is still accruing. While an attractive option, there are specific anatomic and physiologic factors to be considered in each individual case.

## INTRODUCTION

The treatment of aortic rupture has significantly evolved since Parmley’s landmark 1958 paper led to an era in which immediate repair was mandated [1]. It is now recognized that there are three categories of patients: those who die at the scene (70-80% of the whole); those who present unstable or become unstable (2-5% of the whole, with a mortality of 90-98%) and those who are hemodynamically stable and are diagnosed 4-18 hours after injury (15-25% of the whole, with a mortality of 25%, largely due to associated injuries) [2]. In addition, early institution of *B*-blockade in stable patients has provided an increased margin of safety [3, 4]. There have also been modifications in operative technique, including an increased use of mechanical circulatory support which reduce (but do not eliminate) the risks of paralysis, end-organ failure and acute cardiac collapse, and have been associated with improved outcomes [5-7].

Amidst this era of improved operative outcomes and medical management, endovascular stent grafts have become a possible third option. In North America, the concept of thoracic endografting, as an extension of abdominal endograft technology, was greatly stimulated by the Stanford group [8, 9]. Their initial primary interest, and indeed the bulk of the literature since, was with atherosclerotic aneurysms. We now know that endografting is an attractive option that can avoid the morbidity of a thoracotomy in patients with multiple injuries, and that it appears to reduce the risk of paralysis [10-15]. As with all invasive procedures, there are specific complications and anatomic considerations that need to be incorporated into the planning of endovascular treatments of traumatic thoracic injuries.

The purpose of this paper is to review diagnosis, initial management and current treatment options in managing traumatic rupture of the descending thoracic aorta in the acute setting with particular emphasis on endovascular approaches.

## MECHANISM

The primary etiology is rapid acceleration/deceleration. The actual mechanisms described have included shear forces applied at the ligamentum arteriosum, acute compression by the diaphragm, torsion of the aorta, acute intravascular hypertension and/or compression of the aorta between the sternum and spine (“osseous pinch”) [16-18]. It is not surprising that the most common mechanisms include motor vehicle crash, followed by motor cycle crash, pedestrian struck, fall and rarely survivors of airplane crashes [19].

## INCIDENCE

Aortic rupture may be second only to head injury as the primary cause of death following blunt trauma. Despite this, accepting that there are approximately 8000 cases/annum in the United States, and given that as many as 85% of victims die at the scene, then only 1000-1500 cases/annum survive to be treated. In one of the largest contemporary series, 274 patients were admitted to 50 institutions over 2 ½ years. If these cases were distributed evenly the average institution would have seen only 2.2 cases/annum [20]. In practice some centers may manage 8-15 cases/annum, while the majority may encounter 1-2 at most. It does appear that the use of seat belts, air bags and chest protectors has resulted in an increased number of patients surviving motor vehicle and cycle crashes with both fewer associated injuries and smaller aortic defects [21, 22].

## DIAGNOSIS

As many as ½ patients with traumatic rupture have normal thoracic physical findings [23]. Scapular and sternal fractures have not been proven to be independently associated with an increased risk of thoracic aortic rupture [23, 24]. The most specific findings include upper extremity pulse deficit or murmur (suggesting at least great vessel injury), interscapular murmur and/or pseudocoarctaion [25-28].

Because physical findings are generally unhelpful, diagnosis is critically dependent on imaging. Plain chest radiograph (CXR) is the primary screening tool for patients who have sustained a severedeceleration injury, but it is generally accepted that between 2-7% of patients with a traumatic rupture have a normal CXR initially [19]. The incidence of the following plain radiographic findings among patients with diagnosed traumatic thoracic aortic rupture is: widened mediastinum, 89%; obscured aortic knob, 82%; loss of paraspinous stripe, 91%; loss of aortopulmonary window (25%) (all of these in conjunction with widened mediastinum) and bronchial depression, 25% [29, 30]. There is debate, however, on how accurately mediastinal widening can actually be determined [31, 32].

Because plain radiographs are felt to be too unreliable to exclude aortic rupture in patients at risk, there has been an increased emphasis on the importance of mechanism in predicting the need for CT angiography [17, 25, 29, 33, 34]. Some centers have adopted these guidelines as routine, but they are certainly not yet standard of care across the nation. Routinely subjecting patients to CT angiography will inevitably raise concerns about contrat allergies and nephropathy, as well as potentially increased radiation exposure. That being said, if the mechanism is suggestive, and if physical findings suggest severe blunt chest force, then there should be a low threshold for further imaging. At the very least, it is reasonable in completely stable patients, to obtain serial chest radiographs to look for signs of change in the mediastinal contours.

CT angiography has at least the sensitivity and specificity of angiography and usually provides all the data necessary to make the diagnosis and treatment plan [35-37]. Should the diagnosis be made and a pelvic view not taken, then a non-contrast CT of the pelvis should be obtained to evaluate the adequacy of the femoral and iliac arteries for access. Currently, if hematoma is noted around the great vessels, their origin or the arch, but no definite injury is seen, we recommend angiography to exclude associated great vessel injury [38, 39]. Angiography is performed predominantly in patients who clinically appear to be suffering hemorrhage from pelvic fractures. The site of pelvic bleeding is controlled first, and then arch angiography is performed [21, 40]. Trans esophageal echocardiography (TEE) has also been used to make the diagnosis, although its sensitivity and specificity of 57-63% and 84-91% are less than that reported with CT angiography [41]. TEE can be performed during emergent laparotomy, can evaluate cardiac function and/or can be used to confirm small defects and differentiate between pre-existing ulcerated plaques and a true aortic injury [41-44]. Intravascular ultrasound (IVUS) has been used to in a similar fashion [45-47]. Both TEE and IVUS can also be used to measure aortic diameters and location of the great vessels, site of injury etc [43, 46, 48].

## INITIAL MANAGEMENT

In stable patients in whom the diagnosis is suspected, usually because of plain radiographic findings, the initial management should be to control the blood pressure with pain medicine (if there are injuries causing hypertension) and short-acting *B*-blockers [2-4]. This has been shown to reduce the risk of rupture in the acute setting. The goal has often been stated as being “a systolic blood pressure less than 120 mm Hg” but it is reasonable to aim for a pressure “less than or equal to what they presented with” [49]. This control should be maintained until aortic rupture is ruled out or until definitive therapy is performed. It should be remembered that in the majority of patients who are hypotensive, the predominant causes are associated injuries that should take priority [50-52].

## OUTCOMES OF ENDOVASCULAR STENTS UTILIZED IN THE TRAUMA SETTING

A number of series have been published which support the notion that endovascular stents, in the setting of traumatic aortic disruption, have low mortality (predominantly related to associated injuries) and essentially no risk of post-procedure paralysis. When reviewing these data, it is important to consider the span of time in which the experience was accrued (as stent technology has changed significantly over the past few years), recognize the difference between the acute (whether defined as within 24 hours of injury or longer period) *vs*. chronic, and to consider what the indications for stent grafting and contra-indications to open repair were. We have selected those series published 2002-2006 (11 reports), comprising 167 patients, the youngest being 16 years of age [11, 12, 53-62]. These series ranged from 5 – 30 cases, over time periods ranging from 1 – 7 years. Average follow up among the 10 series with at least one year follow up was 24 months. Virtually all stents were industry made, although they varied from ‘dedicated” thoracic stents to a variety of cuff extenders. There were 7 (4%) deaths, two of which were procedure related (one collapse and rupture, one stroke). Type I endoleak occurred in eight instances (4.7%), two healing spontaneously, six requiring further stenting and/or balloon dilation. There were two iliac ruptures reported, and three (1.7%) cases of acute stent collapse requiring operative intervention. There were two cases of non-fatal stroke and one of brachial occlusion requiring thrombectomy. There were no reports of post-procedure paralysis. Dunham and colleagues noted that in patients with isolated chest injuries the length of stay in the intensive care unit and total hospitalization was as low as 1 and 7 days respectively [61]. Lin *et al.* summarized 33 papers describing 324 cases managed endovascularly with follow up ranging from 6-55 months [63]. Technical success was achieved in all but two cases, and there was only one case of paraplegia reported.

These experiences, in combination with an overall major non-fatal complication rate (excluding endoleak) of 4.3% and mortality of 3.6% justifies the excitement that endovascular approaches have provoked in the management of traumatic aortic rupture. Endovascular repair is particularly attractive in managing patients whose associated injuries or comorbid conditions put them at greater risk for open repair [64]. In addition, endografts may also be used not as a definitive repair, but in complicated cases as a “bridge” to definitive treatment in selected patients who are not suitable candidates for either operative repair or medical management [53].

## COMPARISON BETWEEN ENDOVASCULAR AND OPEN REPAIR

Tang and associated presented the results of a meta-analysis comparing the 30-day outcomes between 278 aortic ruptures managed surgically *vs*. 355 managed by endovascular means [65]. There were no significant differences in injury severity or age between the groups. The endovascular group had significantly lower mortality (7.6% *vs*. 15.2%, p = 0.008), paraplegia (0% *vs*. 5.5%, p < 0.0001) and stroke (0.81% *vs*. 5.1%, p=0.003) compared to the open surgical repair cohort [65].

It is inherently difficult to retrospectively compare two techniques that are not necessarily applied to the same patient population with respect to risk assessment, operative experience and institutional biases. Each center has sufficiently different patient populations and management strategies to make it difficult to make broad generalizations based on an individual study. Recognizing that this is not a complete review of all available works, we reviewed eight papers, published between 2004-2008, that specifically compared outcomes within their respective institutions between the two approaches [12, 21, 54, 58, 66-69]. A total of 161 patients underwent open repair. There were 24 deaths (14%) and 5 (3%) cases of new post-operative paralysis. One hundred and sixty-one patients underwent endovascular repair, with 13 (8%) mortality and no new paralysis/paraplegia reported. Only one death was procedure related among the stent graft group (acute stent collapse).

These small comparisons demonstrate that when feasible, endovascular repair appears to be associated with a markedly lower paralysis rate than open repair, that length of stay may be reduced compared to open repair, but that acute outcome is probably more related to overall injury severity than approach. It should also be stressed that the outcome of surgery (as with endovascular repair) is critically linked to initial presentation [21]. With good technique, although mortality rates depending on associated injuries still range from 8-20%, paralysis can be less than 5% [21, 70, 71]. In addition, we do have excellent long term follow up on patients who have undergone open repair, data which is still accruing in the endovascular population [72, 73].

## ENDOVASCULAR REPAIR *VS*. MEDICAL MANAGEMENT

Institution of strict “anti-impulse” therapy should occur once the diagnosis of aortic rupture is suspected [37, 58]. The “ideal” blood pressure depends upon the patient’s age and presenting blood pressure. Until recently, the goal was a systolic blood pressure of < 120 mm Hg and/or mean arterial pressure < 60-70. More recently it has been argued that a blood pressure of “less than what the patient was admitted with” may be more appropriate [2, 49]. When strict blood pressure control is implemented, in stable patients, the risk of rupture in the first week may be as low as 5% or less [2]. Some series have noted improved outcomes with both delayed open and endovascular repair, but this may reflect some selection bias [58]. Reasons for delaying operative intervention include severe head injury, blunt cardiac injury, solid organ injury and/or acute lung injury [6, 74]. In these instances, we have favored serial surveillance imaging (usually with CTA) every 48 hours for 7-10 days, to detect any change in the size or character of the lesion [3]. While the natural history of residual psuedoaneurysms appear to follow those of non-traumatic atherosclerotic aneurysms, these lesions should not, especially in young patients, be considered completely benign, and we favor early intervention as soon as medically stable.

Tight medical control of blood pressure may not be possible in every case. Many patients require other interventions, and monitoring and controlling blood pressure during these can be difficult. There are some hazards including renal and splanchnic insufficiency, and secondary brain injury especially in the setting of increased intra cranial pressure [75]. Although there is some controversy as to the value of driving up cerebral perfusion pressure, or assuming that an increased pressure translates to improved cerebral perfusion, there is general consensus that “high” pressure is associated with a lower risk of secondary brain injury [76-79]. Thus, closed head injury associated with evidence of increased intracranial pressure (by CT and/or ICP monitoring) may actually mandate operative or endovascular repair. One significant advantage of endovascular repair over both operative and non-operative management is that after the stent is placed, it most cases it is possible to allow blood pressure to normalize, or even increase without the risk of bleeding or rupture. We caution that the risk of rupture, even with serial CT angiography and tight hemodynamic control, is not zero. It does appear that after approximately seven days, a persistent psuedoaneurysm follows a natural history more akin to non-traumatic aneurysms, perhaps secondary to inflammation around the injury site [3]. Endovascular stents may be ideally utilized exactly in these patients who cannot under go open operative repair because of significant comorbidities.

The extent of injury may also impact the choice between medical and endovascular management. Minor aortic injuries, involving only small intimal defects, often heal without residual defects [80, 81]. However, even small lesions can go onto to rupture if blood pressure is not controlled [3]. Thus, if blood pressure can be reasonably controlled, and there are no contraindications to medical management, small intimal defects should be managed medically with close follow up. Even small psuedoanerysms, in some cases, have healed [3]. Thus, while endovascular management appears to be an ideal solution in patients with significant co-morbidities, and who are judged to be at too high risk for prolonged medical management, it is not clear that this approach is better than medical management in patients with minimal injuries. One simple guideline is that if the lesion is minimal enough such that one would not consider open operative repair, than one should not rush to endovascular repair either.

## ENDO-GRAFTS CURRENTLY AVAILABLE

The characteristics of an endograft designed for the thoracic aorta, as opposed to the abdominal aorta, include a long enough delivery system to reach the distal arch from the femoral artery, and flexibility accommodate the curvature of the arch. There are variations between different types of grafts in how they deploy, whether or not proximal and/or distal components are bare, whether or not they contain hooks, and how they are actually released from the constraining devices. In general, there has been a shift away from deploying devices in aortic trauma (and type B dissection) which rely on uncovered proximal landing zones because of concerns of aortic perforation [82]. An important consideration is that the average young trauma patient has an aortic diameter in the 20 mm range, which is too small for these devices which were designed for older, atherosclerotic, aortas [83]. Secondly, the use of endografts in the acute trauma setting is considered to be “off label” by the Federal Drug Administration (FDA) outside of trials. A variety of thoracic endografts are available, ranging from 23-46 mm diameter. They are deployed through sheaths ranging from 20-24 Fr. [56, 57, 66, 84-86];Lin, 2007 #131; Wheatley, 2006 #50; Orend, 2002 #26].

Because of the size constraints in the “typical” trauma patient, and because the arch is often acutely angled, some groups have used abdominal aortic cuff extenders rather than dedicated thoracic aortic stent grafts [59, 62, 87]. These are not only smaller, but may actually fit the aortic configuration of transected aortas better, albeit at the expense of needing multiple grafts, of an increased risk of Type III endoleak, and of having to use the shorter delivery system that is designed for the infra-renal aorta [39]. On occasion a contralateral limb or iliac extender from abdominal aortic set may fit the specific anatomic requirements.

## ANATOMICAL CONSIDERATIONS

Anatomic considerations are listed in Table **1**. The initial factor to determine is the diameter of the proximal and distal landing zones. Measurements are taken from inner wall to inner wall. The diameter can be difficult to assess in the distal arch, but one method is to measure the transverse diameter at its widest point. In younger adults the aorta is relatively uniform through the arch.

Because most transections occur in proximity to the left subclavian, the next decision is whether to cover the subclavian origin or not. This may be required in up to 1/3 or more of cases [65, 83]. Deploying the graft within the distal curve (“grey zone”) of the arch may result in partial occlusion of the aorta, increase the risk of stent migration and/or collapse, and result in an endoleak.

The proximal aortic landing zone and arch needs to be reviewed to assess for the presence of significant thrombus and/or calcification. Focal areas of calcification can result in elevating a “lip” of endograft, resulting in increased risk of proximal endoleak. Significant thrombus increases the risk of stroke and distal embolization.

The length of the aorta that needs to be covered is based on a minimum of a 2-cm landing zone. If using cuff extenders, usually three will be required to provide stability [39, 62]. In practical terms, in a number of cases a 2-cm proximal landing zone is not achievable.

Having chosen the optimal size and type of endograft, the next consideration is the length of the delivery device. Commercial thoracic endograft delivery systems have sufficient length to reach the entire thoracic aorta from the femorals, but cuff extenders have delivery systems of only 61-cm that may not reach from the groin to the arch. Additionally, the quality and diameter of the proposed access arteries need to be evaluated. The diameter, angulation and degree of calcification should be determined. Calcifications are better seen with non-contrast images. A non-calcified vessel may tolerate a slightly oversized sheath, but a severely calcified vessel may not accept a sheath that would be predicted to fit based on size criteria alone.

Coverage of the left subclavian artery origin the question of arm ischemia, vertebral-basilar insufficiency and/or type II endoleak. Critical arm ischemia is rare, affecting less than 2% of patients, and if it occurs can be managed electively in most cases [11, 85,  88-90]. Type II endoleak arising by back flow into the pseudoaneurysm is also uncommon as most tears arise from the inner curve. Should Type II endoleak occur, or if there is concern regarding prior to the procedure, the left brachial artery can be accessed and once the graft is deployed, the subclavian can be coiled or closed with a peripheral closure device [91]. Vertebral steal phenomenon can also be addressed electively [92]. Patients with patent left internal mammary grafts should undergo carotid-subclavian bypass prior to left subclavian coverage [88, 92].

Impeding the flow to the left vertebral may pose a risk of posterior cerebellar circulatory insufficiency or stroke. Manninen and associates, based on an autopsy study of 92 deceased patients found that covering the left subclavian would put 5 (5.4%) of patients at risk for posterior stroke due to variation in posterior circulation and right vertebral anatomy [93]. In our experience this has never happened in the younger population, but we are concerned in older patients with diffuse vascular disease. Assessing cerebral circulation is clearly difficult under emergent conditions. Anatomic assessments can be made by CT-angiography or MRA of the head and neck, or cerebral angiography either prior to placement or at the time. We have found transcranial doppler (TCD) to be a useful adjunct. If the basilar artery and the posterior communicating artery can be seen, then flow from the both vertebral arteries, the basilar arteries and posterior communicating arteries can be measured while temporally occluding the origin of the left subclavian artery with an occlusion balloon. Demonstrating intact vertebral-basilar flow upon left subclavian occlusion precludes the need for prophylactic subclavian bypass or transposition [94].

Carotid-subclavian bypass is generally well tolerated, but some investigators have noted an increased stroke risk when this is performed in patients with atherosclerotic aneurismal disease [92, 95]. This may be related to the increased degree of calcification in this older population, and may not apply to the younger trauma patient. Our bias is to attempt to assess cerebral circulation as best we can, but if the case is emergent, and there is no gross evidence of diffuse calcification, we will cover the subclavian if needed without waiting for further imaging, and if subsequent vertebral-basilar or arm ischemia results, to treat this electively.

If the proximal landing zone is felt to encroach upon the origin of the left subclavian, but that complete coverage is not required, it is possible to access the left brachial artery and leave a wire in the arch, which allows precise placement of the device and can permit stenting of the subclavian origin if narrowing occurs [57]. 

## FEMORAL, ILIAC AND AORTIC ACCESS

Access vessel choice depends upon the size of sheath required for the chosen endograft, length of delivery system, quality and diameter of the arteries, and clinical setting. Most trauma patients have healthy vasculature, and thus slight mismatch can be tolerated between sheath size and femoral diameter as long as there is no “tugging” and the sheath advances easily under fluoroscopy. On the other hand, a significant number of trauma patients are young, and have femoral and external iliac arteries that are smaller than 8 mm in diameter, which makes accessing them with the sheaths required for the dedicated thoracic devices problematical. If there is any concern, a contra-lateral sheath should be placed so that balloon occlusion can be used in the event of an iliac rupture during sheath removal. Endografts such as the TAG have been advanced without using the sheath (“bare back”) but this is not recommended because the graft can catch on an edge and deploy prematurely or be damaged. When withdrawing the sheath at the end of the case, particularly if a percutaneous approach has been used, it is critical that the blood pressure be monitored for two to three minutes as any acute drop is pathognomonic of an iliac rupture.

Retroperitoneal iliac exposure may be required if using cuff extenders and the device is not long enough to reach the location of the tear and/or if the femoral arteries are too small and/or calcified to use. If there has been pelvic trauma, using the side with the least hematoma is desirable. The common iliac can be accessed directly or a 10 mm silo graft is anastomosed end-to-side. If the pelvis is deep, to avoid a problem with angulation, the silo graft can be tunneled through the lower abdominal soft tissue or indeed through the femoral canal to the groin. Patients who have had prior aorto-iliac grafts represent can be challenging because the iliacs are often imbedded in scar tissue. The ureter should always be mobilized anteriorly, avoiding dissection on both sides to prevent devascularization. In the vast majority of cases the best that can be achieved is that enough dissection of the iliac limb of the graft allows application of a partial occlusion clamp or direct graft puncture. Having completed the procedure, whether anastomosing to graft or native vessel, the conduit is simply truncated and over sewn as a patch. In some circumstances it may be advisable to convert the conduit to an ilio-femoral artery bypass. This allows a relatively easier access route for later percutaneous interventions should the need arise.

Some patients may already have an open abdomen, and in these cases direct infra-renal aortic access can be used [39]. This would not be a good choice if there has been visceral spillage.

## FOLLOW UP

The protocols for follow up are based on the various clinical trials designed predominantly to evaluate thoracic endografting for atherosclerotic aneurysms. Typical guide-lines include CT angiography at 48 hours, discharge, 1-,6- and 12-months and then annually. These protocols are designed to detect graft collapse, migration or persistent endoleak with aneurysmal growth. To a large extent, these guidelines were laid out because the cases involved patients with diseased landing zones with a potential for ongoing dilation of the aorta. Obvious concerns include following patients with renal insufficiency, as well as the burden of a large number of radiation exposures. Patients with renal insufficiency can be surveyed with IVUS, TEE, MRA or even CT without contrast. The primary concern is whether or not there is psuedoaneurysm regression or growth. Simple chest radiography can detect stent deformation or migration. For aortic transaction cases we tend to obtain a CT angiography at 48 hours, at one month, at one year and then follow with chest radiographs. When obtaining a CT angiogram, it is important to make sure that the study is performed in a uniform manner: triphasic with unenhanced, enhanced and delayed images.

We have not used antiplatelet agents for thoracic aortic stent grafting. However, we treat these like any other implant, and recommend antibiotic prophylaxis for any invasive procedure (such as dental work).

## COMPLICATIONS

### Endoleak

In brief, endoleak can be categorized as Type I (leak around the proximal, A, or distal, B, ends of the graft), II (leak form an artery feeding into the aneurysm sac), III (leak between components) or IV (failure of graft integrity). The forms of most concern in the trauma setting are proximal Type I and to some degree type II endoleaks. Proximal Type I endoleaks occur in approximately 5% of cases. Persistent type I endoleak is associated with a risk of late rupture [84]. The predominant mechanism in trauma patients is the combination of a short landing zone and lack of apposition along the inner curvature of the arch [96]. Gentle ballooning should be tried first. If this is not sufficient, then extending proximally with another graft should follow [57, 97]. Type I leaks that are visualized only on delayed images immediately following deployment may resolve following heparin reversal. We assess these at 48 hours with a repeat CT angiogram. Blood pressure should be controlled with *B*-blockade during this period. Proximal Type I endoleaks found on follow up imaging can be usually managed by repeat interventions, including using proximal cuff extenders [69]. Significant leaks seen at the time of implant, or at follow up, that do not respond to further ballooning or extension should under go operative repair. Most type I endoleaks occur within 30 days, but occasionally can be found up to 2 years later, reinforcing the need for strict surveillance [96]. 

Type II endoleaks should be managed based on whether or not the left subclavian is the source. If it is, coil occlusion of the subclavian, carotid to subclavian bypass with proximal ligation or carotid subclavian to carotid transposition should be performed. Left subclavian arterial causes are of Type II endoleak are less common in the trauma setting than the more typical atherosclerotic aneurysm case where there is circumferential dilation and the subclavian is more likely to feed into the aneurysm. Type II endoleaks believed to be secondary to patent bronchial or intercostal arteries are more common, but again are less common in the trauma setting as there are fewer branches in the proximal descending thoracic aorta. Some investigators believe that these are more benign than in the setting of abdominal endografting, and that in the majority of cases they will seal spontaneously [98]. Rarely a branch vessel can be accessed and coiled using microcatheter techniques.

### Stent Graft Collapse

This is a catastrophic complication that can occur immediately, or within the first 48 hours, but it has been seen up to three months post procedure [98-102]. It is felt that this represents a combination of graft over sizing and a lack of apposition along the inner curve of the aorta. In younger patients without significant calcification, over sizing should be in the 7-15% range rather than the 20% range used for managing atherosclerotic aneurysms [71, 73]. In young hyperdynamic aortas, with their degree of pliability, the force of the cardiac ejection that hits the under side of the graft causes collapse of the graft [63, 101, 103]. This usually leads to immediate aortic occlusion and possibly rupture. If this occurs post implant, the patient will develop signs of acute coarctation, and rapid onset of paralysis and renal failure can occur. This may not be immediately apparent if the patient is still on the ventilator and sedated. Prevention includes very accurate sizing, choosing a graft that approximates a 10% over sizing rather than 20%. It is also important to plan pre- and intra-operatively to avoid landing in the “no man’s land” of the aorta. If the proximal portion of the graft is not apposed or at least close to the inner curve, particularly if there is only a short zone of apposition, perhaps less than 50%, then options include extending the graft proximally and/or repeat ballooning [99]. Uncovered bare metal stents deployed within the stent graft have also been used both acutely and when collapse occurs in a delayed fashion. There has been some concern that these bare stents may either erode over time through the graft fabric or create proximal aortic perforations [82]. There is not enough data to determine the real risk of this occurring, but theoretically a short bare stent will conform more closely to the aortic curvature than a bare proximal portion that is secured to an endograft and has reduced flexibility. Across the country there have been numerous anecdotal reports of bare stent extenders being used for proximal partial or complete collapse with good short term results. There is growing consensuses that perhaps cuff extenders, which may be deployed sequentially and thus fit the curvature of the aorta better, may prove to perform better than longer thoracic stent grafts in patients with aortic diameters smaller than 24 mm.

If stent graft collapse occurs post-operatively, it can often be detected by plain chest radiography, or by non-contrast CT. Immediate intervention is required. If complete collapse has occurred, explanting and operative repair is prudent, but ballooning and extending the device with a bare stent has been used with success [62]. Anectodotally, axillary-femoral-femoral bypass has been used as a temporizing measure, but ultimately the stent must be removed.

### Dissection/Rupture

Free rupture can occur at any time. Prevention is strict blood pressure control, particularly during periods of transfer or other procedures that might acutely elevate the heart rate and/or blood pressure. At the time of initial wire passage, great care should be given to watching the wire advance. If there is difficulty negotiating the aortic curvature or there is narrowing at the injury site, a directional catheter such as a vertebral and/or hydrophilic catheter can be invaluable.

There have been cases of delayed or immediate rupture after the graft has been deployed. Endografts which feature a bare metal proximal extension have been implicated in perforating the aortic wall [104]. Even covered grafts which do not have this feature have been implicated if there is poor apposition of the aortic wall with resultant motion against the wall. All three dedicated thoracic endografts discussed here have been implicated in acute or delayed perforation with/or without dissection, at least anecdotally, in the non-trauma experience. Proper graft sizing is essential to facilitate good graft-aortic apposition.

Proximal dissection has also been documented. One mechanism is that during ballooning of the proximal cuff, the ends of the graft can create a dissection flap that rapidly progresses retrograde. To avoid this, initial ballooning should be a gentle as possible, just enough to document profiling of the balloon along the side of the graft Ballooning should only be done within the graft. Late psuedoaneurysm development has also been recorded and attributed to injury to the aortic wall during stent deployment [68]. Presumably, this is due to a similar mechanism.

### Migration

If the proximal landing zone is not long enough, and the aneurysm itself is large, stents can migrate distally. This may be detected on routine chest radiograph, or may present with a new endoleak. In younger patients, as aortic growth occurs, an endograft may loose its fixation. If this should occur, options include both operative explanting and grafting, or proximal extension with another endograft. This is one of the reasons that life long surveillance is necessary.

## THE PEDIATRIC AND ADOLESCENT PATIENT

Endovascular aortic stent grafts are not commonly used in pediatric and adolescent patient [105]. These patients usually have aortas that are too small for currently available devices, will likely grow and the lack of long term durability has greater significance in this population. In particular, growth of the native aorta may predispose the patient to later migration risks [63]. Thus, while endovascular approaches may be considered as a bridge to definitive treatment, at this time surgery is still considered the standard for children and youths.

## FUTURE TRENDS

Endograft technology is continuing to evolve, but perhaps even more significantly, experience and longer follow up data is beginning to accrue as well. Branched grafts are beginning to be designed for both arch and abdominal visceral vessels. Specific for the trauma population, a variety of grafts which are shorter, precurved and smaller are being developed which will allow more precise deployment and potentially reduce in complication rates.

## CONCLUSION

Endovascular repair of the traumatically injured thoracic aorta has emerged as an exceptionally promising modality that is typically quicker than open repair, with a reduced risk of paralysis. There are a specific set of anatomic criteria that need to be applied, which can be rapidly assessed by the CT angiogram. The enthusiasm for endovascular repair must be tempered by recognition of the complications and lack of long term follow up, particularly in younger patients. Surgeons who are skilled in open aortic repair must not only be involved, but should take on a leadership role during the planning, deployment and follow up of these patients. Familiarity with all of the available devices expands treatment options. As more specific devices become available, and more follow up is accrued, the role of endovascular stents will continue to grow.

## Figures and Tables

**Table 1:** Anatomic Considerations

Anatomical Features to Consider	Implications
Diameter of proximal and distal landing zones	Determines size of endograft that can/should be utilized
Distance from lesion to origin of Left Subclavian Artery	Will obtaining an adequate landing zone require coverage of the Left Subclavian Artery?
Distance from lesion to origin of Left Common Carotid Artery	If required, is there room to land distal to the origin of the Left Common Carotid Artery? Will there be room, if needed, to clamp distal to the origin or will circulatory arrest be needed if subsequent operative repair is needed?
Degree of curvature across the proximal landing zone	Is there a high likelihood that to avoid malposition along the inner curvature that the graft will have to placed more proximally?
Quality of the aorta	Is there significant thrombus and/or calcification that would pose a risk of stroke or type I endoleak?
Quality of access vessels	Is the diameter sufficient to permit the required sheath? Are the more proximal calcifications and/or tortuosity that might prevent safe passage of the sheath?
Distance from proposed access vessel to the lesion	Does the system being used have sufficient length to reach the proposed site?
Length of the injury	If using cuffs, how many may be required to ensure fixation
Vascular anomalies	Anomalous origin of Left vertebral Artery? Patent LIMA graft? Aberrant origin of Right Subclavian Artery?
